# Structural and sociodemographic determinants of BREAST-Q outcomes after gender-affirming breast augmentation in transgender women

**DOI:** 10.1016/j.jpra.2026.05.011

**Published:** 2026-05-08

**Authors:** Daniel S. Rouhani, Matthew F. McLaughlin, Nina Mehta, Dominick Falcon, Daniel Soroudi, Zuivanna Rivas, Nathan Ramrakhiani, Esther A. Kim

**Affiliations:** a School of Medicine, University of California San Francisco, San Francisco, CA, USA; b School of Medicine, University of North Carolina, Chapel Hill, NC, USA; c Division of Plastic and Reconstructive Surgery, Geisinger Medical Center, Danville, PA, USA; d Department of Surgery, Division of Plastic and Reconstructive Surgery, University of California San Francisco, San Francisco, CA, USA

**Keywords:** Gender-affirming surgery, Breast augmentation, Patient-reported outcomes, BREAST-Q, Health disparities

## Abstract

**Purpose:**

Sociodemographic factors are associated with disparities in postoperative outcomes after gender-affirming surgery, yet their relationship with patient-reported outcomes after gender-affirming breast augmentation remains unclear. We evaluated associations between patient-level and structural factors and patient-reported outcome measures (PROMs) among transgender women after breast augmentation.

**Methods:**

Patients who underwent gender-affirming breast augmentation from 2015 to 2023 at two affiliated institutions, a county safety-net hospital and an academic medical center, were recruited to complete a postoperative BREAST-Q Augmentation Module survey ≥ 12 months after surgery. Six subscales were analyzed. Univariate linear regression was used to assess associations between sociodemographic, clinical, and surgical factors and BREAST-Q scores.

**Results:**

Among 270 eligible patients, 65 (24 %) completed the BREAST-Q ≥ 1 year post-augmentation. Surgeries performed at the county hospital correlated with lower satisfaction with breasts (β = -15.45; *p* = 0.032), psychosocial well-being (β = -15.46; *p* = 0.042), and physical well-being (β = -14.23; *p* = 0.002) compared to procedures performed at the academic setting. Current tobacco use at the pre-operative visit was associated with lower postoperative psychosocial well-being (β = -23.78; *p* = 0.028). Severe obesity (BMI ≥ 35 kg/m²) was linked to lower implant satisfaction (β = -1.66; *p* = 0.013). Black patients reported higher sexual well-being compared with non-Hispanic White patients (β = +25.67; *p* = 0.016). No significant associations were found between other sociodemographic factors, surgical variables (implant size, shape, texture, incision), complications, or race/ethnicity for the remaining BREAST-Q subscales.

**Conclusion:**

After gender-affirming breast augmentation, patient-reported satisfaction and well-being among transgender women undergoing breast augmentation appear to be shaped more by structural context and patient-level factors rather than by the technical factors of the operation alone.

## Introduction

Gender-affirming care is recognized by the World Professional Association for Transgender Health (WPATH) Standards of Care (version 8), as medically necessary for many transgender and gender-diverse patients to alleviate gender dysphoria and improve quality of life.^1^^,^^2^ An estimated 1.6 million people in the United States identify as transgender, and many experience gender dysphoria related to incongruence between their physical characteristics and gender identity.^3^ For some transgender women, breast development is a central component of their transition and can significantly enhance psychosocial comfort and confidence.^4^^,^^5^ Despite hormone therapy, up to 70% of transgender women pursue breast augmentation to achieve desired breast size and appearance.^6^^,^^7^ Prior studies report high satisfaction after gender affirming breast surgery and improvement in psychosocial and sexual well-being, with reductions in gender dysphoria.^8^, ^9^, ^10^, ^11^, ^12^, ^13^

Emerging evidence suggests that these benefits are not experienced equally across patients. Shamamian et al.^14^ found that Black patients undergoing gender-affirming genital surgery experienced disproportionately higher complication rates, longer hospitalizations, and greater costs compared with White patients. Trilles et al.^15^ similarly identified higher odds of 30-day complications, readmission, and reoperation among Black transgender patients. Jolly et al.^16^ further demonstrated that patients experiencing socioeconomic and structural barriers to care had higher rates of systemic complications. Collectively, these findings highlight the influence of social and structural determinants on outcomes in gender-affirming surgery.

Patient-reported outcome measures (PROMs) are essential for evaluating the impact of gender-affirming interventions on quality of life and for guiding patient-centered care.^17^^,^^18^ The BREAST-Q, a widely validated PROM, measures patient satisfaction and quality of life after breast augmentation, reduction, and reconstruction.^19^, ^20^, ^21^ In cisgender populations, studies have examined racial and ethnic differences in BREAST-Q scores following breast procedures. Kim et al.^22^ found that Asian, Black, and Hispanic patients reported lower scores than White patients after implant-based reconstruction, while Hanson et al.^23^ reported similarly lower scores in American Indian/Alaska Native patients following postmastectomy breast reconstruction (PMBR). These findings underscore that social and structural determinants can influence PROMs, but their effects may vary across procedures and patient populations.

Although PROMs are well validated in cisgender breast surgery, their use in gender-affirming breast augmentation remains limited, and the impact of sociodemographic and structural factors on postoperative satisfaction in transgender women is not well defined. To address this gap, the present study evaluates the association between these factors and BREAST-Q outcomes in transgender women undergoing breast augmentation.

## Methods

### Study design and population

The study was approved by our Institutional Review Board (#14–14,439). This cross-sectional study included patients who underwent gender-affirming breast augmentation between 2015 and 2023 at two affiliated institutions: a county safety-net hospital and an academic medical center. Eligible participants were adults (≥18 years) who underwent primary bilateral gender-affirming breast augmentation and were at least 12 months postoperative at the time of recruitment. Patients were excluded if the BREAST-Q survey was incomplete or could not be completed due to language barriers. Sociodemographic, clinical, and perioperative variables were abstracted from the electronic health record.

### Sociodemographic characteristics

Demographic variables collected included age at the time of survey (<40 vs. ≥40 years), race/ethnicity (Non-Hispanic White, Hispanic, Black/African American, or Other), and housing stability. Substance use variables collected included tobacco recorded at the pre-operative visit (current, former, or never) and alcohol use (yes/no). Clinical variables included body mass index (BMI) categories (<30 kg/m², 30–34.9 kg/m², and ≥35 kg/m²) and medical comorbidities. Information on prior gender-affirming surgeries was collected, categorizing patients as having undergone no bottom surgery, orchiectomy, or vaginoplasty. Hormone therapy data included median length of hormone treatment and median time living as the identified gender.

### Surgical characteristics and outcomes

Surgical variables included the hospital setting (academic medical center vs. county hospital), surgical approach (subglandular vs. dual plane), and incision type (periareolar vs. inframammary). Implant characteristics were recorded, including implant size (≥450 mL vs. <450 mL), shape (round vs. anatomic), and texture (smooth vs. textured). Postoperative complications were recorded and categorized as surgical complications (e.g., infection, hematoma) or aesthetic complications (e.g., asymmetry, implant malposition, or capsular contracture).

### Patient-reported outcome measures with BREAST-Q

The BREAST-Q postoperative augmentation module (version 2.0), a validated tool for measuring patient-reported outcomes in breast surgery, was used to measure patient satisfaction and well-being.^19^, ^20^, ^21^ Although the BREAST-Q was developed and validated in cisgender breast surgery cohorts, it was utilized here as a standardized breast augmentation specific PROM with established scoring and interpretability, allowing our results to be benchmarked against the BREAST-Q breast augmentation literature. The GENDER-Q was developed after this study began and therefore was not incorporated into the survey instrument. Six subscales were evaluated: satisfaction with breasts, satisfaction with implants, satisfaction with outcome, physical well-being, psychosocial well-being, and sexual well-being. All subscales except satisfaction with implants were converted from their raw values to the standard 0‑100 BREAST‑Q scale, with higher scores indicating greater satisfaction or well‑being. The satisfaction‑with‑implants scale is a two‑item stand‑alone measure, consequently, only its raw totals (0–8) were analyzed. Patients were recruited by phone and email to complete the BREAST-Q questionnaire at least 12 months postoperatively to ensure assessment of long-term satisfaction and surgical outcomes.

### Statistical analysis

Demographic and clinical characteristics were summarized using means (standard deviations) for normally distributed continuous variables and counts (percentages) for categorical variables. Where appropriate, medians (interquartile ranges) were used for skewed data. Differences in BREAST-Q subscale scores were examined across patient subgroups defined by age (> 40 vs. ≤ 40 years), BMI classification, race/ethnicity, institutional site (county vs. academic medical center), and surgical characteristics. Linear regressions were conducted to determine whether specific patient or clinical factors were associated with differences in BREAST-Q subscale scores. To assess whether hospital setting might confound these associations, sociodemographic and surgical variables were also compared by hospital site using chi-squared analyses. For variables that differed by hospital site, exploratory linear regression models adjusting for hospital setting were performed to evaluate whether these factors were associated with BREAST-Q outcomes independent of site. All analyses were exploratory in nature, intended to describe potential associations rather than test adjusted or causal effects. Statistical significance was set at p < 0.05.

## Results

### Sociodemographic and clinical characteristics

Among the 270 eligible patients, 65 (24%) completed the postoperative survey at least one year after surgery. The sample was racially and ethnically diverse: almost half were Non‑Hispanic White (47.7 %), while 21.5 % were Hispanic, 13.9 % Black or African American, and 16.9 % reported another race or ethnicity. Participants were evenly split by age, with 49.2% under 40 and 50.8% aged 40 or older. Most respondents (80%) reported stable housing, and 61.5% were non-tobacco users, with 46.2% reporting current alcohol use. 67.7% of participants had a BMI below 30 kg/m². 50.8% reported at least one medical comorbidity, most commonly obesity (30.8%), hypertension (20.3%), or HIV infection (14.5%). Few had undergone prior genital gender-affirming surgery, and the median duration of hormone therapy was 45 months. Participants had been living as their identified gender for a median of 4 years (Table 1).

**Table 1:** Sociodemographic, clinical, and preoperative characteristics of patients who completed > 1 year post-op BREAST-Q survey.

Variable	Total (n = 65) n (%)
**Age at time of survey**	
< 40 years	32 (49.2)
≥ 40 years	33 (50.8)
**Race/ethnicity**	
Non-Hispanic White	31 (47.7)
Hispanic	14 (21.5)
Black or African American	9 (13.9)
Other	11 (16.9)
**Housing status**^a^	
Stably housed	52 (80.0)
Unstably housed	5 (7.7)
Unknown	8 (12.3)
**Tobacco use**^a^	
No	40 (61.5)
Current	7 (10.8)
Former	17 (26.2)
Unknown	1 (1.5)
**Alcohol use**^a^	
Yes	30 (46.2)
No	35 (53.9)
**BMI (kg/m^2^)**^a^	
< 30 kg/m^2^	44 (67.7)
≥ 30 kg/m^2^ and < 35kg/m^2^	12 (18.5)
≥ 35 kg/m^2^	8 (12.3)
Unknown	1 (1.5)
**Any medical comorbidity**	33 (50.8)
**Specific medical comorbidities**	
Obesity	20 (30.8)
HIV	9 (14.5)
Hypertension	13 (20.3)
Asthma	9 (14.1)
Diabetes	5 (7.8)
CAD	6 (9.4)
CVA/Stroke	2 (3.1)
Liver disease	0 (0.0)
Kidney disease	2 (3.1)
IBD	1 (1.6)
COPD	0 (0.0)
**Prior bottom surgery**^a^	
None	48 (73.9)
Orchiectomy	9 (13.9)
Vaginoplasty	8 (12.3)
**Median length of hormone treatment (months)**^a^^,^*(IQR)	45 (24–66)
**Median time living as identified gender (years)**^a^^,^⁎⁎**(IQR)**	4 (2–9)

a Collected at time of surgery

⁎ Missing values (n = 63, missing n = 2)

⁎⁎ Missing values (n = 62, missing n = 3)

### Surgical characteristics

Most procedures (75.4%) were performed at an academic center, the rest (24.6%) at a county hospital. Subglandular pockets were utilized in 61.5% of cases and dual‑plane pockets in 38.5%. Inframammary incisions predominated (92.3%) and implants ≥450 cc were selected in 60% of procedures. Round (78.5%) and smooth (76.9%) implants were more common over anatomic (21.5%) and textured (23.1%) implants. Complications occurred in 30.8% of cases, mainly aesthetic (27.7%) rather than surgical (6.2%) (Table 2).

**Table 2:** Surgical characteristics of patients who completed > 1 year post-op BREAST-Q survey.

Variable	Total (n = 65) n (%)
**Hospital setting**	
Academic center	49 (75.4)
County Hospital	16 (24.6)
**Surgical approach**	
Subglandular	40 (61.5)
Dual plane	25 (38.5)
**Incision**	
Periareolar	5 (7.7)
Inframammary	60 (92.3)
**Implant size**^a^	
≥ 450cc	39 (60.0)
< 450cc	26 (40.0)
**Implant shape**	
Anatomic	14 (21.5)
**Implant texture**	
Smooth	50 (76.9)
Textured	15 (23.1)
**Any complication**	20 (30.8)
Surgical^b^	4 (6.2)
Aesthetic^c^	18 (27.7)

a Patients grouped based on largest unilateral implant

b Includes hematoma, periprosthetic infection, implant extrusion, superficial surgical site infection, wound dehiscence, nipple necrosis, and permanent numbness

c Includes implant asymmetry, malposition/rotation, capsular contracture, symmastia, hypertrophic scarring, animation deformity, implant rippling, and residual tuberous breast deformity

### Factors associated with BREAST-Q subscale outcomes

**Fig. 1:**
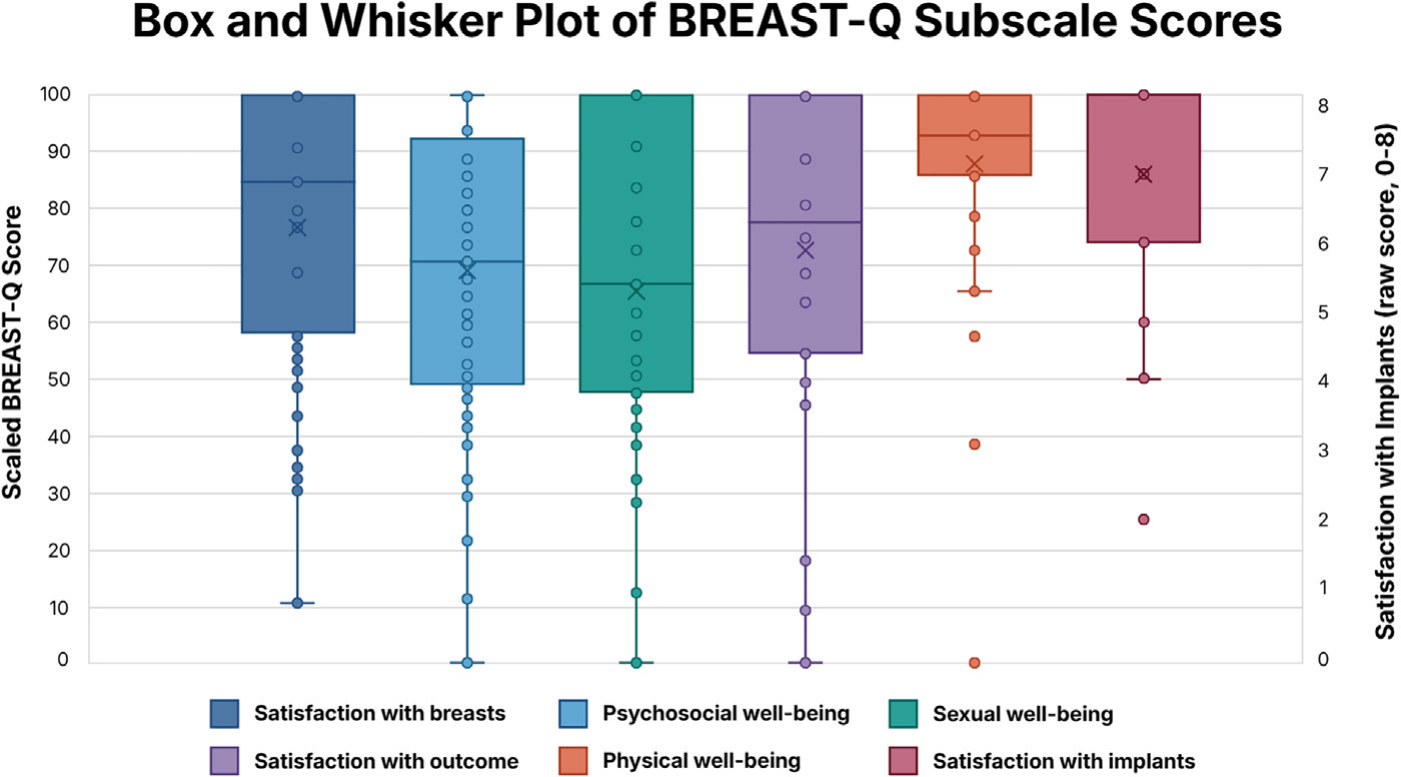
Box‑and‑whisker plot of BREAST‑Q sub‑scale scores ≥ 1 year after gender‑affirming breast augmentation (n = 65). Box‑and‑whisker plots show the interquartile range (boxes), median (horizontal bar), mean (x), and range excluding outliers (whiskers). Individual patient scores are overlaid as circles. All subscales except Satisfaction with Implants are presented on a 0–100 scale (left y-axis), where higher scores indicate greater satisfaction or well-being. Satisfaction with Implants is a two-item subscale reported as raw scores ranging from 0 to 8 and is displayed on a separate right-sided y-axis.

Overall BREAST-Q scores demonstrated high satisfaction across all six subscales (Fig. 1). Being a Black or African American patient was associated with higher sexual well-being scores compared with Non-Hispanic White patients (β = 25.67; p = 0.016). Tobacco use at the time of pre-operative consultation was correlated with diminished psychosocial well-being (β = -23.78; p = 0.028). BMI ≥ 35 kg/m² was linked to lower satisfaction with implants (β = -1.66; p = 0.013) compared to BMI < 30 kg/m². Among sociodemographic factors, housing stability, alcohol use, prior bottom surgery, and duration of hormone treatment or time living as one’s identified gender showed no significant associations with BREAST-Q subscales (all p > 0.05) (Table 3). Patients undergoing surgery at the county hospital were associated with reduced satisfaction with breasts (β = -15.45; p = 0.032), psychosocial well-being (β = -15.46; p = 0.042), and physical well-being (β = -14.23; p = 0.002). Surgical variables, including implant size, shape, texture, incision type, and complications (surgical or aesthetic), were not significantly associated with any BREAST-Q subscale (Table 4).

**Table 3:** Linear regression between sociodemographic factors and BREAST-Q subscale scores among patients who completed > 1 year post-op BREAST-Q survey.

Variable	BREAST-Q Subscales											
	Satisfaction with breasts		Satisfaction with implants		Satisfaction with outcome		Physical well-being		Psychosocial well-being		Sexual well-being	
	β	p	β	p	β	p	β	p	β	p	β	p
**Age at time of survey**												
< 40 years	Reference		Reference		Reference		Reference		Reference		Reference	
≥ 40 years	-8.27	0.187	-0.03	0.944	-6	0.383	3.95	0.34	-0.87	0.896	-12.91	0.064
**Race/ethnicity**												
Non-Hispanic White	Reference		Reference		Reference		Reference		Reference		Reference	
Hispanic	-1.23	0.883	-0.15	0.793	0.01	0.999	-9.36	0.083	2.08	0.812	11.87	0.183
Black	0.68	0.944	-0.27	0.689	-7.02	0.513	0.84	0.893	10.82	0.293	25.67	**0.016**
Other	0.4	0.965	0.88	0.159	-2.58	0.794	-2.57	0.658	1.57	0.869	9.04	0.351
**Housing status**^a^												
Unstably housed	Reference		Reference		Reference		Reference		Reference		Reference	
Stably housed	-4.82	0.691	-0.68	0.35	1.08	0.936	-8.26	0.277	-13.77	0.275	-13.27	0.314
**Tobacco use**^a^												
No	Reference		Reference		Reference		Reference		Reference		Reference	
Current	-15.74	0.126	0.6	0.412	-16.42	0.15	-4.66	0.498	-23.78	**0.028**	-11.58	0.322
Former	6.06	0.4	0.46	0.367	-0.82	0.918	1.14	0.815	0.22	0.977	1.41	0.863
**Alcohol use**^a^												
No	Reference		Reference		Reference		Reference		Reference		Reference	
Yes	-0.31	0.96	-0.59	0.184	-2.7	0.697	-4.83	0.244	-1.44	0.829	3.38	0.634
**BMI (kg/m^2^)**^a^												
< 30kg/m^2^	Reference		Reference		Reference		Reference		Reference		Reference	
≥ 30 kg/m^2^ and < 35kg/m^2^	6.98	0.402	0.42	0.445	7.23	0.43	7.41	0.168	8.08	0.362	0.16	0.987
≥ 35 kg/m^2^	-5.9	0.547	-1.66	**0.013**	-6.15	0.569	-8.84	0.164	-0.5	0.962	-0.6	0.958
**Any medical comorbidity**												
No	Reference		Reference		Reference		Reference		Reference		Reference	
Yes	0.89	0.888	0.15	0.732	-2.12	0.761	2.76	0.508	6.35	0.342	-1.68	0.813
**Prior bottom surgery**^a^												
None	Reference		Reference		Reference		Reference		Reference		Reference	
Vaginoplasty and/or orchiectomy	-6.22	0.385	-0.02	0.967	-10.14	0.195	-0.81	0.864	-4.68	0.537	-8.53	0.287
**Length of hormone treatment prior to surgery (months)**^a^^,^*(IQR)												
< 24 months	Reference		Reference		Reference		Reference		Reference		Reference	
≥ 24 months	-4.54	0.532	-0.75	0.141	-3.5	0.659	-2	0.678	-1.63	0.832	5.24	0.522
**Length of time living as identified gender prior to surgery (years)**^a^^,^⁎⁎**(IQR)**												
< 4 years	Reference		Reference		Reference		Reference		Reference		Reference	
≥ 4 years	2.71	0.693	0.31	0.491	5.97	0.428	2.43	0.569	3.74	0.606	10.57	0.161

Bold indicates statistical significance (p < 0.05)

β) Signifies regression coefficient

a Collected at time of surgery

⁎ Missing values (n = 63, missing n = 2)

⁎⁎ Missing values (n = 62, missing n = 3)

**Table 4:** Linear regression between surgical variables and BREAST-Q subscale scores among patients who completed > 1 year post-op BREAST-Q survey.

Variable	BREAST-Q Subscales											
	Satisfaction with breasts		Satisfaction with implants		Satisfaction with outcome		Physical well-being		Psychosocial well-being		Sexual well-being	
	β	p	β	p	β	p	β	p	β	p	β	p
**Hospital setting**												
Academic Center	Reference		Reference		Reference		Reference		Reference		Reference	
County Hospital	-15.45	**0.032**	-0.52	0.311	-14.22	0.073	-14.23	**0.002**	-15.46	**0.042**	-11.58	0.155
**Surgical approach**												
Dual plane	Reference		Reference		Reference		Reference		Reference		Reference	
Subglandular	-2.36	0.717	-0.74	0.1	0.71	0.921	0.39	0.927	1.37	0.842	-0.675	0.926
**Incision**												
Periareolar	Reference		Reference		Reference		Reference		Reference		Reference	
Inframammary	-5.67	0.632	-0.63	0.445	-5.72	0.66	-4.55	0.559	-7.72	0.537	-7.13	0.59
**Implant size**^a^												
< 450 mL	Reference		Reference		Reference		Reference		Reference		Reference	
≥ 450cc	3.97	0.537	-0.49	0.279	8.38	0.233	4.46	0.291	1.17	0.864	-1.94	0.788
**Implant shape**												
Round	Reference		Reference		Reference		Reference		Reference		Reference	
Anatomic	-1.25	0.87	-0.2	0.708	1.08	0.898	2.69	0.593	0.56	0.945	-2.08	0.809
**Implant texture**												
Smooth	Reference		Reference		Reference		Reference		Reference		Reference	
Textured	0.82	0.913	-0.11	0.839	3.93	0.679	3.03	0.538	3.19	0.686	-1.32	0.875
**Any complication**	-3.64	0.594	0.56	0.244	-7.83	0.294	-3.03	0.5	-2.13	0.768	-3.71	0.628
**Any surgical complication**^b^	-12.81	0.328	-0.02	0.986	-9.03	0.53	0.43	0.961	-0.03	0.998	4.62	0.753
**Any aesthetic complication^c^**	-3.52	0.617	0.59	0.228	-9.14	0.235	-3.52	0.447	-4.38	0.556	-7.18	0.362

Bold indicates statistical significance (p < 0.05)

β) Signifies regression coefficient

a Collected at time of surgery

### Differences in characteristics by hospital setting

Because hospital setting was associated with several BREAST-Q outcomes, we examined whether selected sociodemographic and surgical characteristics differed by site (analyses not shown). Among the sociodemographic variables evaluated, age and race/ethnicity were not associated with hospital setting, whereas housing status differed significantly by site (p = 0.038), with unstable housing being more common at the county hospital. Among surgical variables, implant shape (p = 0.016) and implant texture (p = 0.012) varied by hospital, with anatomic and textured implants more frequently used at the academic center. Implant size, incision type, and implant placement approach did not differ by site. In exploratory regression models adjusting for hospital site, surgical factors remained unassociated with BREAST-Q outcomes, consistent with the unadjusted analyses. In contrast, unstable housing was associated with lower physical well-being only after adjustment for hospital site, raising the possibility of confounding by treatment setting.

## Discussion

This study is among the first to examine how sociodemographic and structural factors relate to BREAST-Q outcomes after gender-affirming breast augmentation in transgender women. We found that, although overall satisfaction and quality-of-life scores were high across domains, outcomes were not uniform. Patients treated at the county safety-net hospital, those with current tobacco use, and those with severe obesity all reported lower satisfaction or well-being, whereas implant characteristics, incision type, and other technical factors of the procedures were not associated with differences in BREAST-Q scores. In addition, Black/African American patients reported higher sexual well-being than Non-Hispanic White patients, underscoring that the impact of race on PROMs is complex and not uniformly disadvantageous. Together, these findings suggest that structural and patient-level factors play a far greater role in shaping patient-reported outcomes than the technical details of the operation alone.

The most striking disparities in our cohort were related to treatment setting. Patients undergoing gender affirming breast augmentation at the county safety-net hospital reported significantly lower satisfaction with breasts, psychosocial well-being, and physical well-being compared with those treated at the affiliated academic medical center. These differences may reflect not only individual patient-level factors but also structural and institutional inequities in the broader U.S. healthcare system.^24^ In a national study of 3096 hospitals Chatterjee et al.^24^ found that safety-net hospitals scored significantly lower than non safety-net hospitals on nearly every domain related to patient reported experiences. Safety-net hospitals caring for uninsured or underinsured populations are also more likely to be penalized under value-based payment systems, limiting their ability to reinvest in quality improvement.^25^ Gonzales et al.^26^ found that transgender and gender-nonconforming adults had higher odds of being uninsured and delaying or avoiding needed care because of cost compared with cisgender adults. Puckett et al.^27^ similarly reported that financial and insurance barriers, limited availability of gender-affirming services, and provider bias or limited knowledge were among the most common obstacles to accessing treatment. These documented barriers likely compound within safety-net settings, where resource limitations and higher rates of uninsurance may further hinder access to affirming care, which can help explain the lower postoperative satisfaction and well-being observed in our safety-net cohort.

An important consideration is the possibility of confounding by differences in patient and surgical characteristics across institutions. In additional analyses, housing status, implant shape, and implant texture differed by hospital setting, whereas age, race/ethnicity, implant size, incision type, and implant placement approach did not. Because some variables varied by site, residual confounding cannot be excluded. However, in models adjusting for hospital setting, surgical factors remained unassociated with BREAST-Q outcomes, supporting our interpretation that technical aspects of the operation were not the primary drivers of between-site differences. In contrast, unstable housing was associated with lower physical well-being only after adjustment for hospital site, indicating that the relationship between housing status and physical well-being may have been obscured when hospital setting was not considered.

Our study also demonstrated that higher BMI (≥35 kg/m²) was associated with lower satisfaction with implants. This may be partly due to a greater likelihood of subglandular implant placement in higher BMI patients, which is more prone to visible rippling.^28^ Additionally, patients with severe obesity may have more constrained implant selection (including limited availability or candidacy for larger-volume implants based on chest width, soft-tissue characteristics, and safety considerations), which may reduce the likelihood of achieving desired breast size and thereby lower implant satisfaction. These results mirror findings in cisgender populations.^29^^,^^30^ A study of 554 cisgender female patients conducted by Jørgensen et al.^29^​ found that higher BMI was significantly associated with lower satisfaction across multiple BREAST-Q domains, including satisfaction with breasts, sexual well-being, and satisfaction with outcome, reinforcing the negative impact of elevated BMI on postoperative satisfaction.

We also found that current tobacco use documented at the preoperative visit was associated with significantly lower postoperative psychosocial well-being. This pattern is consistent with a variety of studies across specialties demonstrating that cigarette smoking is linked to worse patient-reported outcomes after surgery.^31^, ^32^, ^33^, ^34^ It is also likely that this association reflects underlying psychosocial and structural factors, as smoking is consistently associated with elevated rates of depression, anxiety, and psychological distress, and is more common among individuals experiencing socioeconomic disadvantages and reduced social support.^31^^,^^33^^,^^35^

In contrast, our finding that Black/African American patients reported higher sexual well-being than Non-Hispanic White patients differs from much of the existing literature in cisgender breast surgery, where racial and ethnic minority patients often report equal or lower BREAST-Q scores than White patients.^29^, ^30^, ^31^, ^32^ For example, in a study of 3281 cisgender women, Kim et al.^22^ found that Black and Hispanic patients had lower physical well-being scores than White patients, while Asian patients scored lower across all BREAST-Q domains, including sexual well-being. Our findings therefore suggest that the relationship between race and PROMs may differ in the context of gender-affirming procedures and is not uniformly disadvantageous for certain groups, however, further research is needed to confirm this association.

Variables such as prior bottom surgery, hormone duration, and time living as one’s identified gender did not significantly affect BREAST-Q scores. While these factors are often linked to broader indicators of social and gender affirmation, their lack of association in this study may reflect either limitations in sample size or the possibility that breast augmentation itself exerts a dominant effect on satisfaction, independent of other transition-related experiences. Most surgical variables, including implant size, type, incision approach, and complications, were not significantly associated with differences in BREAST-Q scores. These findings suggest that psychosocial factors may have a greater influence on patient satisfaction than specific technical aspects of the surgery.^36^, ^37^, ^38^

There are several limitations to this study. The sample size was relatively small, and only a quarter of eligible patients completed the survey, raising concerns about response bias. Patients who are more satisfied or have greater engagement with the healthcare system may have been more likely to participate, potentially skewing results. Moreover, this study relied solely on univariate linear regression, which does not adjust for potential confounding variables. Larger studies should incorporate multivariable analyses to better isolate independent predictors. Additionally, the BREAST-Q, while validated for breast surgery, was developed for cisgender populations, and its sensitivity to the unique needs and experiences of transgender individuals may be limited.

Future studies should consider incorporating the recently developed GENDER-Q,^39^ a patient-reported outcome measure designed specifically for gender-affirming care. Informed by input from transgender and gender-diverse individuals across multiple countries, the GENDER-Q includes 55 scales covering domains such as psychosocial well-being, body image, sexual health, and experience of care. Future research should also aim to replicate these findings in larger, multicenter cohorts and explore additional factors, such as mental health history, discrimination, and social support, that may influence patient satisfaction and well-being.

## Conclusion

In this cohort of transgender women undergoing gender-affirming breast augmentation, treatment at a county safety-net hospital was associated with lower BREAST-Q scores for satisfaction with breasts, psychosocial well-being, and physical well-being compared with treatment at an affiliated academic medical center. Severe obesity (BMI ≥ 35 kg/m²) and current tobacco use were also linked to lower satisfaction and psychosocial well-being, whereas implant characteristics, incision type, and other technical aspects of the operation were not associated with differences in patient-reported outcomes. Black/African American patients reported higher sexual well-being than Non-Hispanic White patients, suggesting that the relationship between race and PROMs in gender-affirming surgery may differ from patterns described in cisgender breast surgery. Taken together, these findings indicate that structural context and patient-level factors may have a greater influence on postoperative satisfaction and well-being than operative details alone and underscore the importance of equity-focused models of gender-affirming care.

## Author contributions

**Daniel Rouhani:** Contributed to the writing of the abstract, introduction, methods, results, conclusion, and references; assisted with data analysis and table preparation; and participated in manuscript editing. Reviewed and approved the final manuscript. **Matthew McLaughlin:** Assisted in study design; contributed to writing the abstract; performed data collection; conducted data analysis; and prepared tables. Provided significant feedback during manuscript development and approved the final manuscript. **Nina Mehta:** Contributed to data collection and database organization. Reviewed and approved the final manuscript. **Dominick Falcon:** Contributed to data collection and database organization. Reviewed and approved the final manuscript. **Daniel Soroudi:** Contributed to data collection and database organization. Reviewed and approved the final manuscript. **Zuivanna Rivas:** Contributed to data collection and database organization. Reviewed and approved the final manuscript. **Nathan Ramrakhiani:** Contributed to data collection and database organization. Reviewed and approved the final manuscript. **Esther Kim:** Assisted in study design; provided significant feedback during manuscript development and approved the final manuscript.

## Ethical approval

This study was approved by the University of California, San Francisco Institutional Review Board (#14-14439).

## Financial disclosure statement

None.

## Declaration of competing interest

None declared.
